# Supplementary material for: Discovery of an agonistic Siglec-6 antibody that inhibits and reduces human mast cells
Source: Commun Biol. 2022 Nov 11;5:1226. doi: 10.1038/s42003-022-04207-w (PMC9652399; doi:10.1038/s42003-022-04207-w)
Supplement: Supplementary file 2 — Supplementary Information [file 42003_2022_4207_MOESM2_ESM.pdf]

SUPPLEMENTARY APPENDIX

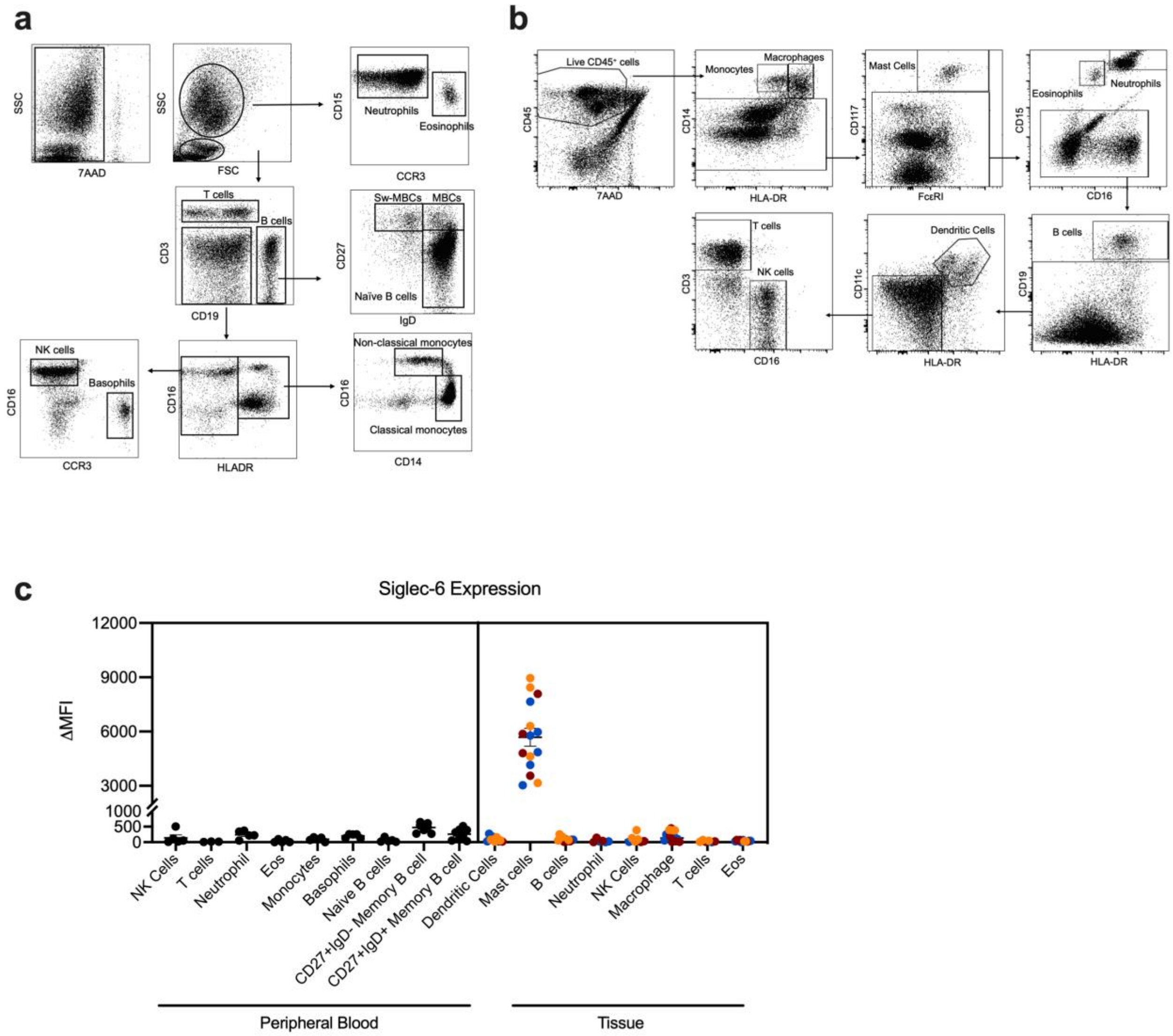

Supplementary Figure 1: Siglec-6 is expressed on human tissue MCs. Flow cytometry gating strategy for immunophenotyping (a) peripheral blood and (b) dissociated human tissues. (c) Cell surface expression of Siglec-6 on immune cells identified in Panels A and B from healthy peripheral blood (n=5-8) and human skin (orange; n=5), lung (red; n=2-4), and GI tissues (blue; n=4-6). Expression is represented as delta median fluorescence intensity (dMFI) and was calculated by subtracting the MFI from a fluorescence minus one negative control. Data are plotted as mean ± SD for individual donors.

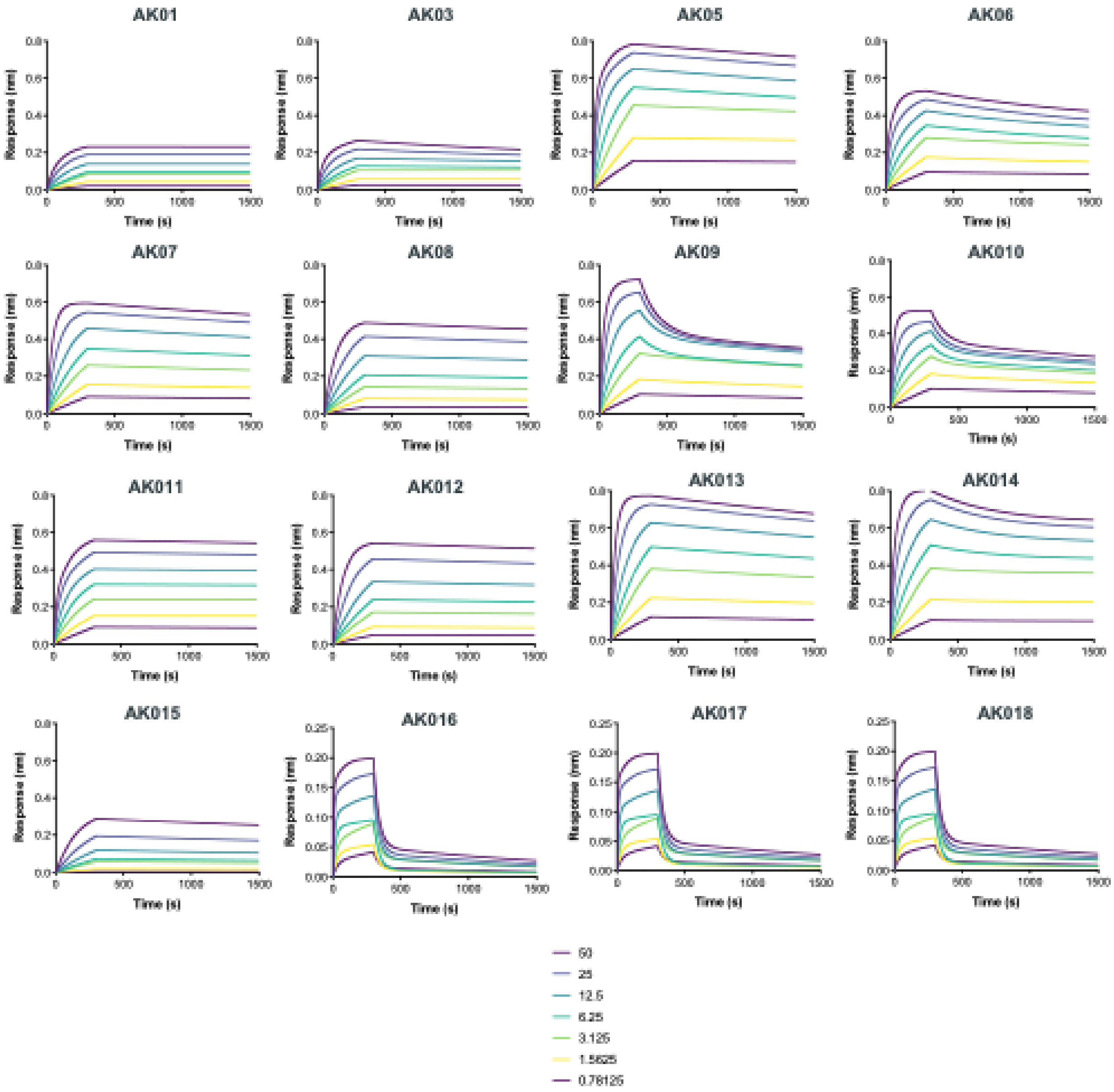

Supplementary Figure 2: Binding properties of Siglec-6 mAbs to Siglec-6. Assessment of Siglec-6 mAb avidity by biolayer interferometry. Representative curves are shown for all Siglec-6 mAbs not shown in Figure 1.

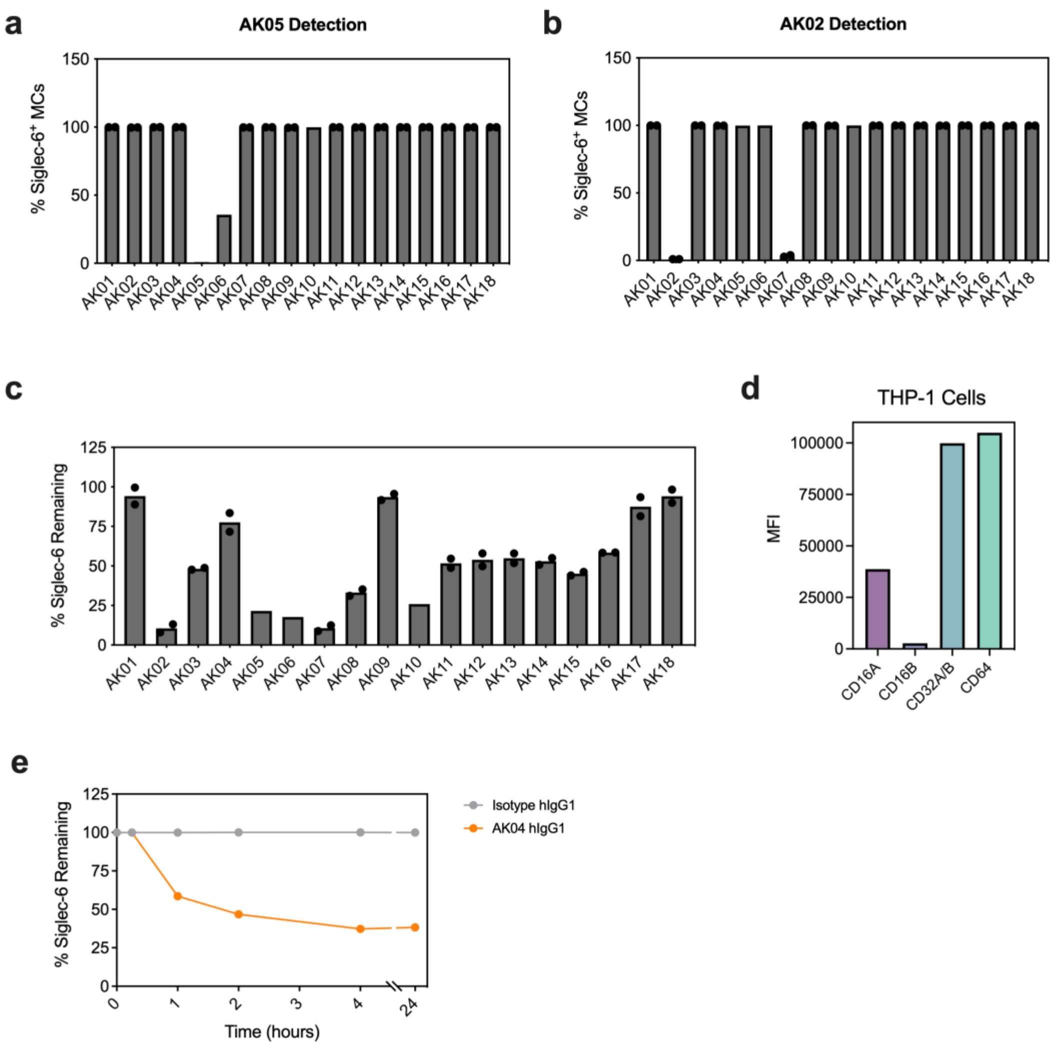

Supplementary Figure 3: Detection and evaluation Siglec-6 receptor internalization. The percentage of Siglec-6+ hMCs (n=2 donors) determined using the detection antibody (a) AK05 or (b) AK02 after 30-minute incubation at 4C with the indicated Siglec-6 mAbs. (c) Siglec-6 internalization on hMCs (n=2 donors) after overnight incubation with the indicated Siglec-6 mAb clones at 5 μg mL^{-1} as determined by flow cytometry using the detection antibody AK05 (for clones AK01-AK04, AK07-AK18) or AK02 (for clones AK05-AK06). (d) Expression of FcγRs on THP-1 cells as determined by flow cytometry. (e) Siglec-6 internalization at 25 minutes, 1, 2, 4, or 24 hours on hMCs (n=1 donor) in the presence of THP-1 cells post-treatment with 5 μg mL^{-1} AK04 (orange) or isotype control mAb (gray). Data are representative of 3 independent experiments. Internalization was normalized to isotype control-treated hMCs for each timepoint with representative donor shown.

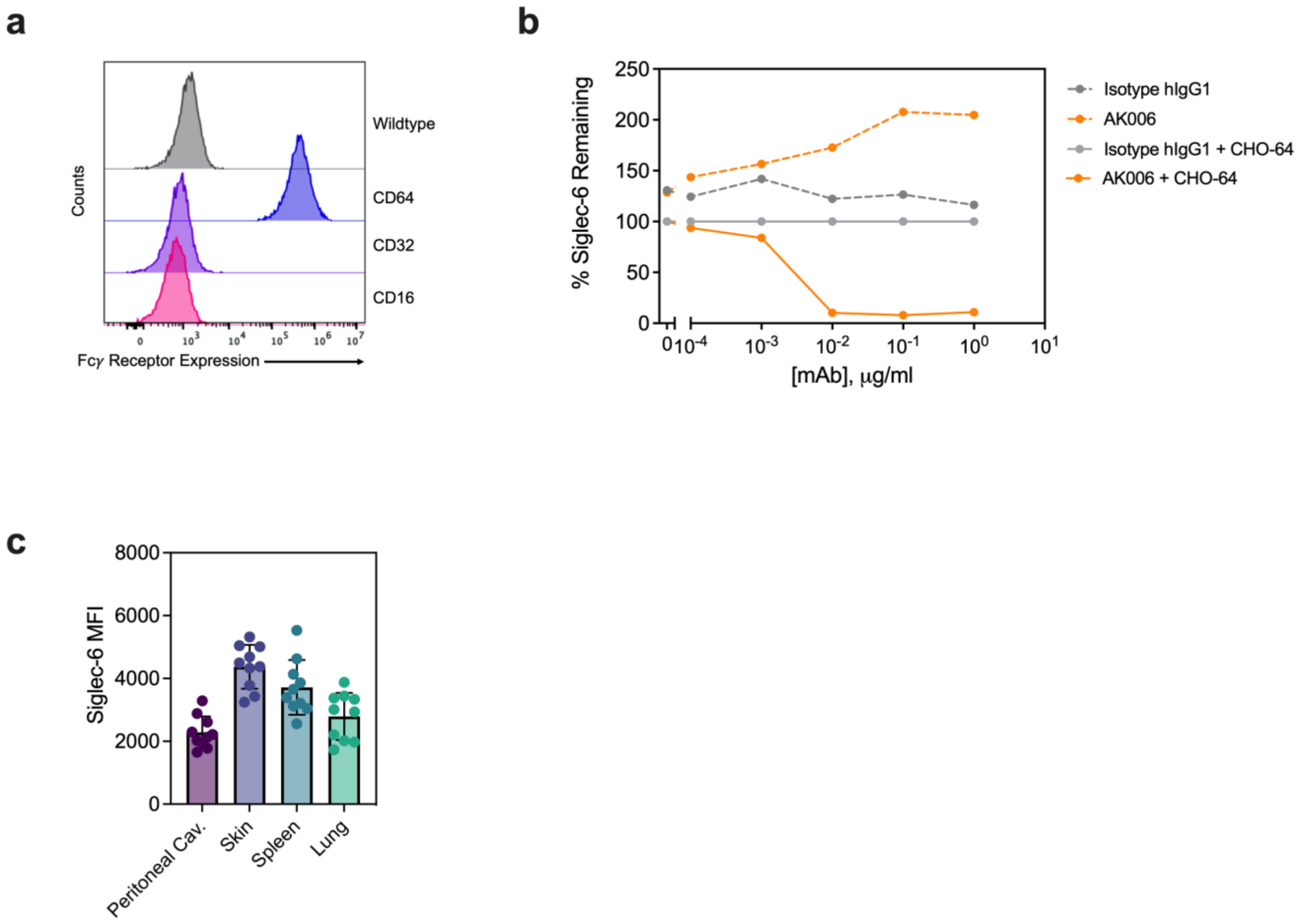

Supplementary Figure 4: AK04-mediated receptor internalization is FcR dependent. (a) Representative histogram of human CD64 expression on CHO-64 cells. (b) Siglec-6 internalization on hMCs (n=1 donor) after incubation with AK04 hIgG1 at various concentrations in the absence (dashed lines) or presence of CHO-64 cells (5:1 CHO to MC ratio) as determined by flow cytometry using the detection antibody AK05. Data are representative of 2 independent experiments. (c) Expression of Siglec-6 on the indicated mast cell populations from humanized mice (mean ± SD; n=10 mice).

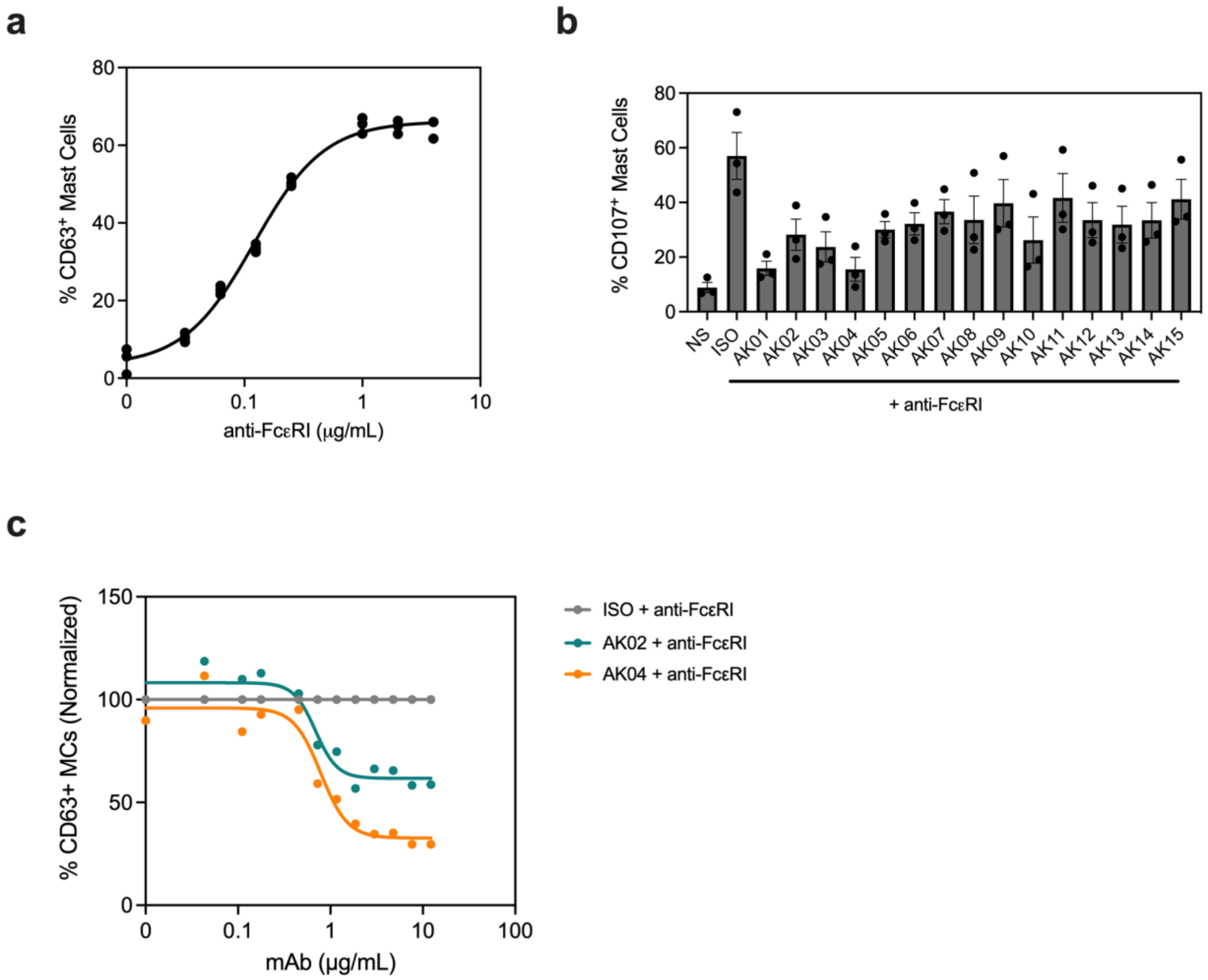

Supplementary Figure 5: Siglec-6 mAbs inhibit IgE-mediated MC activation. (a) Percentage of CD63+ hMCs (n=3 donors) after 20-min incubation with the indicated concentration of anti-FcεRI antibody (CRA-1). (b) Percentage of CD107a+ hMCs (n=3 donors) non-stimulated or activated with anti-FcεRI antibody (CRA-1, 250 ng mL^{-1}) in the presence of Siglec-6 mAb clones (5 µg mL^{-1}) as determined by flow cytometry. (c) Percentage of CD63+ hMCs activated with anti-FcεRI antibody (CRA-1, 250 ng mL^{-1}) in the presence of titrating concentrations of AK02 (green) or AK04 (orange) as determined by flow cytometry. Data are representative of 3 independent experiments.

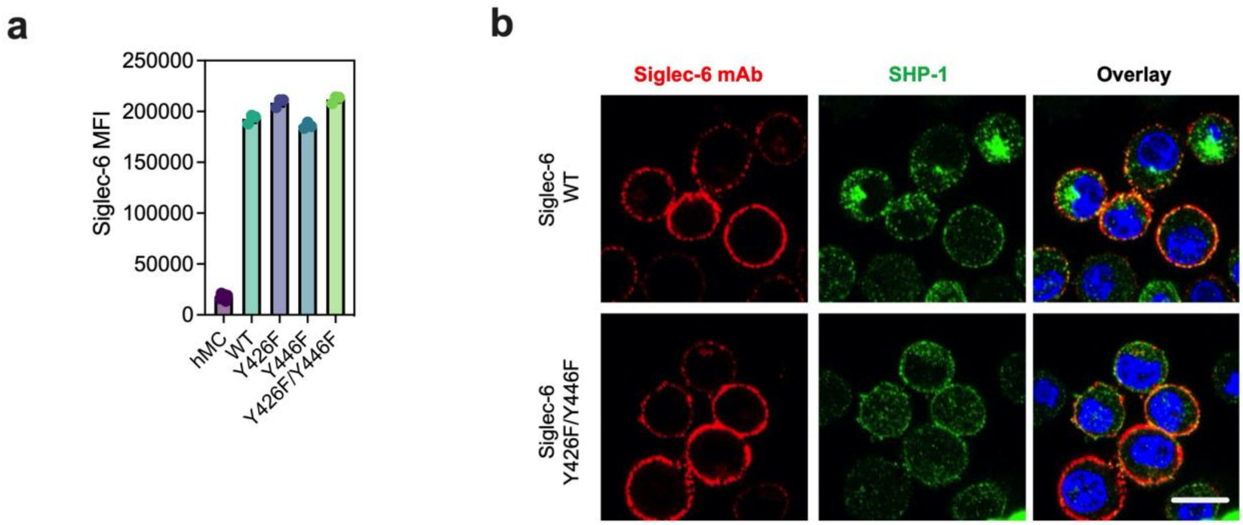

Supplementary Figure 6: Siglec-6 expression on BMMCs and co-localization with Shp-1. (a) Expression of Siglec-6 on hMCs (n=6 donors) and transiently transfected BMMCs with WT and ITIM mutant constructs (n=3 donors) as determined by flow cytometry. (b) Confocal images of BMMCs transfected with WT or double mutant Siglec-6 constructs, treated with an anti-Siglec-6 mAb (red) for 0 minutes (unstimulated), and stained with an anti-Shp-1 antibody (green). Data are plotted as mean ± SEM for Panel A. Scale bar represents 10μM.

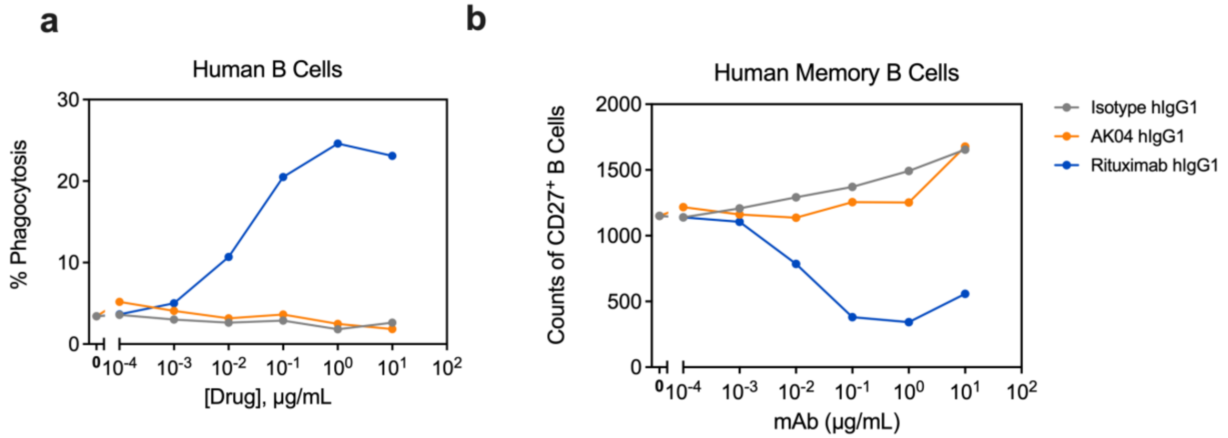

Supplementary Figure 7: Rituximab, but not AK04 induces ADCP of B cells. (a) Percentage of phagocytosis of human CD19+ B cells after 4-h incubation with THP-1 cells in the presence of titrating concentrations of rituximab (blue), AK04 hIgG1 (orange), or an isotype hIgG1 control (gray). (b) Absolute counts of memory B cells (CD19+ CD27+) after 4-h incubation with THP-1 cells in the presence of titrating concentrations of rituximab (blue), AK04 hIgG1 (orange), or an isotype hIgG1 control (gray). Data are plotted as mean percentage or counts (n=1 donor) and are representative of 2 experiments.

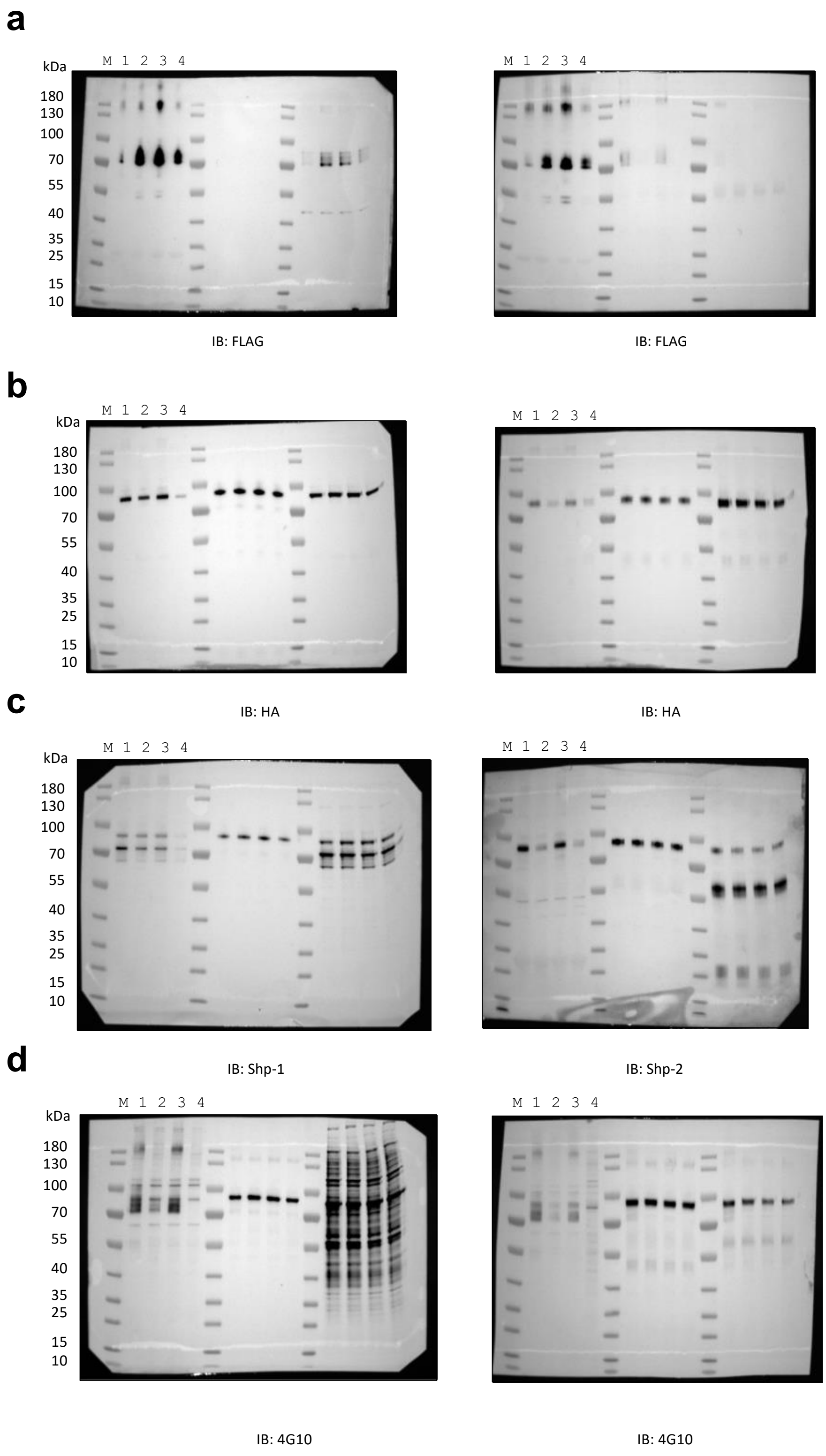

Supplementary Figure 8: Uncropped western blots for Figure 4c. Uncropped blots are shown for (a) panel a, (b), panel b, (c) panel c, and (d) panel d.
