# Supplementary material for: Discovery of an agonistic Siglec-6 antibody that inhibits and reduces human mast cells
Source: Commun Biol. 2022 Nov 11;5:1226. doi: 10.1038/s42003-022-04207-w (PMC9652399; doi:10.1038/s42003-022-04207-w)
Supplement: Supplementary file 3 — Description of Additional Supplementary Files [file 42003_2022_4207_MOESM3_ESM.pdf]

Description of Additional Supplementary Files

File name: Supplementary Data 1

Description: Source data for graphs in paper

File name: Supplemental Video 1

Description: Video of AK04 and AK02-induced Siglec-6 internalization
