# Supplementary material for: Discovery of an agonistic Siglec-6 antibody that inhibits and reduces human mast cells
Source: Commun Biol. 2022 Nov 11;5:1226. doi: 10.1038/s42003-022-04207-w (PMC9652399; doi:10.1038/s42003-022-04207-w)
Supplement: Supplementary file 6 — Reporting Summary [file 42003_2022_4207_MOESM6_ESM.pdf]

nature portfolio

Corresponding author(s): Bradford A Youngblood

Last updated by author(s): Oct 11, 2022

Reporting Summary

Nature Portfolio wishes to improve the reproducibility of the work that we publish. This form provides structure for consistency and transparency in reporting. For further information on Nature Portfolio policies, see our Editorial Policies and the Editorial Policy Checklist.

Statistics

For all statistical analyses, confirm that the following items are present in the figure legend, table legend, main text, or Methods section.

n/a	Confirmed	
☐	☒	The exact sample size (*n*) for each experimental group/condition, given as a discrete number and unit of measurement
☐	☒	A statement on whether measurements were taken from distinct samples or whether the same sample was measured repeatedly
☐	☒	The statistical test(s) used AND whether they are one- or two-sided<br>*Only common tests should be described solely by name; describe more complex techniques in the Methods section.*
☒	☐	A description of all covariates tested
☐	☒	A description of any assumptions or corrections, such as tests of normality and adjustment for multiple comparisons
☒	☐	A full description of the statistical parameters including central tendency (e.g. means) or other basic estimates (e.g. regression coefficient) AND variation (e.g. standard deviation) or associated estimates of uncertainty (e.g. confidence intervals)
☐	☒	For null hypothesis testing, the test statistic (e.g. *F*, *t*, *r*) with confidence intervals, effect sizes, degrees of freedom and *P* value noted<br>*Give P values as exact values whenever suitable.*
☒	☐	For Bayesian analysis, information on the choice of priors and Markov chain Monte Carlo settings
☒	☐	For hierarchical and complex designs, identification of the appropriate level for tests and full reporting of outcomes
☒	☐	Estimates of effect sizes (e.g. Cohen's *d*, Pearson's *r*), indicating how they were calculated

Our web collection on statistics for biologists contains articles on many of the points above.

Software and code

Policy information about availability of computer code

Data collection	NA
Data analysis	NA

For manuscripts utilizing custom algorithms or software that are central to the research but not yet described in published literature, software must be made available to editors and reviewers. We strongly encourage code deposition in a community repository (e.g. GitHub). See the Nature Portfolio guidelines for submitting code & software for further information.

Data

Policy information about availability of data

All manuscripts must include a data availability statement. This statement should provide the following information, where applicable:

- Accession codes, unique identifiers, or web links for publicly available datasets
- A description of any restrictions on data availability
- For clinical datasets or third party data, please ensure that the statement adheres to our policy

Source data underlying main figures are provided in Supplementary Data 1. Uncropped and unedited images of the blots that appear in the main article are provided in Supplementary Data 2. Allakos materials described in this manuscript may be available to qualified academic researchers upon request. In certain circumstances in which we are unable to provide a particular proprietary reagent, an alternative molecule may be provided that behaves in a similar manner.

Human research participants

Policy information about studies involving human research participants and Sex and Gender in Research.

Reporting on sex and gender	NA
Population characteristics	NA
Recruitment	NA
Ethics oversight	NA

Note that full information on the approval of the study protocol must also be provided in the manuscript.

Field-specific reporting

Please select the one below that is the best fit for your research. If you are not sure, read the appropriate sections before making your selection.

☒ Life sciences ☐ Behavioural & social sciences ☐ Ecological, evolutionary & environmental sciences

For a reference copy of the document with all sections, see nature.com/documents/nr-reporting-summary-flat.pdf

Life sciences study design

All studies must disclose on these points even when the disclosure is negative.

Sample size	sample size was determined using standard practices for in vitro and in vivo mouse experiments; certain individual experiments using humanized mice were small so multiple experiments were pooled as noted in relevant figure legends
Data exclusions	No data was excluded
Replication	attempts at replication were successful
Randomization	mice were randomized into treatment groups based on body weights
Blinding	investigators were blinded to antibody treatments for in vivo experiments

Reporting for specific materials, systems and methods

We require information from authors about some types of materials, experimental systems and methods used in many studies. Here, indicate whether each material, system or method listed is relevant to your study. If you are not sure if a list item applies to your research, read the appropriate section before selecting a response.

Materials & experimental systems

n/a	Involved in the study	
☐	☒	Antibodies
☐	☒	Eukaryotic cell lines
☒	☐	Palaeontology and archaeology
☐	☒	Animals and other organisms
☒	☐	Clinical data
☒	☐	Dual use research of concern

Methods

n/a	Involved in the study	
☒	☐	ChIP-seq
☐	☒	Flow cytometry
☒	☐	MRI-based neuroimaging

Antibodies

Antibodies used	Anti-FLAG (Sigma), Anti-HA (Sigma), Anti-SHP1/SHP2 (Cell Signaling, Clone D50F2), Anti-pTyr (Millipore, Clone 4G10), Anti-FceRI (Biolegend, MAR-1), Anti-CD11b (M1/70), HLA-DR (340549), Anti-CD45 (368528), Anti-CD117 (130091733), Anti-CD14 (301834), Anti-CD15 (555401), Anti-CD16 (557744), Anti-CD3 (OKT3), Anti-CD1c (301606), Anti-CD19 (302208), Anti-Siglec-6 (R&D Systems, MAB2859), anti-CD20 (302330), Anti-CD38 (303532), Anti-CD27 (356410), anti-IgD (348210), Anti-Siglec-6 mAbs (Allakos), Isotype Control mIgG1 and hIgG1 (Allakos, MOPC1). Anti-CD63 (Biolegend, NVG-2)
Validation	All antibodies were validated via manufacturer's website or internal assays

Eukaryotic cell lines

Policy information about cell lines and Sex and Gender in Research

Cell line source(s)	THP-1 monocytic cell line (ATCC)
Authentication	none
Mycoplasma contamination	confirmed no mycoplasma
Commonly misidentified lines (See ICLAC register)	none

Animals and other research organisms

Policy information about studies involving animals; ARRIVE guidelines recommended for reporting animal research, and Sex and Gender in Research

Laboratory animals	Humanized mice engrafted with CD34+ human stem cells (NSG-SGM3) were purchased from Jackson Laboratories
Wild animals	NA
Reporting on sex	NA
Field-collected samples	NA
Ethics oversight	Samples and data from animals were collected in accordance with the National Research Council's Guide for the Care and Use of Laboratory Animals

Note that full information on the approval of the study protocol must also be provided in the manuscript.

Flow Cytometry

Plots

Confirm that:

☒ The axis labels state the marker and fluorochrome used (e.g. CD4-FITC).

☒ The axis scales are clearly visible. Include numbers along axes only for bottom left plot of group (a 'group' is an analysis of identical markers).

☒ All plots are contour plots with outliers or pseudocolor plots.

☒ A numerical value for number of cells or percentage (with statistics) is provided.

Methodology

Sample preparation	Approximately 1–5×106 cells were preincubated with CD16/32 antibody to block nonspecific binding. Cells were then incubated at 4°C for 10 min with staining antibody panels, washed, and fixed in 2% paraformaldehyde.
Instrument	NovoCyte flow cytometer (Penteon model)
Software	FlowJo
Cell population abundance	all cell populations evaluated were found in abundance
Gating strategy	specifically shown in Supplemental figure 1

☒ Tick this box to confirm that a figure exemplifying the gating strategy is provided in the Supplementary Information.
